# Dual *Ureaplasma parvum* arthritis: a case report of *U. parvum* septic arthritis following contralateral reactive arthritis in an immunosuppressed patient

**DOI:** 10.1186/s12879-021-06733-0

**Published:** 2021-10-29

**Authors:** Lea Lemoine, Cecile Le Brun, Francois Maillot, Camille Thorey, Annaelle Boucaud, Adrien Lemaignen, Adrien Bigot

**Affiliations:** 1 Department of Infectiology, University Hospital of Tours, 2 Boulevard Tonnellé, Tonnellé, France; 2 Department of Internal Medicine and Clinical Immunology, University Hospital of Tours, 2 Boulevard Tonnellé, Tonnellé, France; 3 Department of Bacteriology-Virology-Hygiene, University Hospital of Tours, 2 Boulevard, Tonnellé, France; 4 UMR INSERM 1253, 10 Boulevard Tonnellé, Tours, France; 5 Faculty of Medicine of Tours, François Rabelais University, 10 Boulevard Tonnellé, Tours, France

**Keywords:** *Ureaplasma parvum*, Reactive arthritis, Septic arthritis, Immunosuppression, Case report

## Abstract

**Background:**

*Ureaplasma parvum* is usually part of the normal genital flora. Rarely can it cause invasive infections such as genitourinary infections, septic arthritis, or meningitis.

**Case presentation:**

Here we present the first description of chronic ureterocystitis in a 56-year-old immunocompromised patient, complicated first by reactive arthritis and secondarily by contralateral septic arthritis due to *U. parvum* infection. *U. parvum* was detected in synovial fluid and in a urine sample. Treatment consisted of double-J stenting and targeted antibiotic therapy. Evolution showed resolution of urinary symptoms and clinical improvement of arthritis despite functional sequelae.

**Conclusions:**

Given the high prevalence of *U. parvum* colonisation, this diagnosis should remain a diagnosis of exclusion. However, because of the difficulty in detecting this microorganism, it should be considered in unexplained subacute urethritis or arthritis, including reactive arthritis, especially in immunosuppressed patients. Real-time PCR positivity in the absence of a differential diagnosis should not be overlooked.

**Supplementary Information:**

The online version contains supplementary material available at 10.1186/s12879-021-06733-0.

## Background

In 1954, Shepard et al., discovered that species from the genera *Ureaplasma*, part of the *Mycoplasmataceae* family together with *Mycoplasma*, were pathogens causing non-gonococcal urethritis [1]. *Ureaplasma urealyticum and Ureaplasma parvum* are the main species identified as pathogenic and were identified in 1999 [2] .

*U. urealyticum* is a common pathogen in genitourinary infections, reactive arthritis [3], and occasionally septic arthritis [4]. In contrast, *U. parvum* is usually considered part of the normal genital flora and therefore non-pathogenic. Nevertheless, it can occasionally be an opportunistic or iatrogenic [5] pathogen and be responsible for genital tract infections [6] or respiratory disease in newborns [7]. Given the prevalence of colonisation in the female genital tract, which is up to 40%[8], *U. parvum* positive screening on multiplex real-time PCR for sexually transmitted infection (STI) is usually not reported because of lack of clinical significance. However, a few reports of *U. parvum* infections, including post-surgical meningitis [4] and septic arthritis but not reactive arthritis, have been described to date.

Here, we report a case of *U. parvum* septic arthritis following reactive arthritis as a complication of genitourinary infection in an immunosuppressed woman.

## Case presentation (see Additional file 1: Timeline)

A 56-year-old woman was admitted in March 2019 for diagnostic work up. She had received treatment for follicular lymphoma with R-CHOP from October 2017 to April 2018 and was still on rituximab. She had no other significant medical history.

She first presented in April 2018 with arthritis of the right knee and received ceftriaxone, then cefpodoxime-proxetil as a probabilistic antibiotic therapy, with favourable outcome. Arthrocentesis was not performed.

In October 2018, the patient reported dysuria and haematuria. Urine tests showed leukocyte count > 1000/mm^3^ and red cell count > 1000/mm^3^, but culture remained sterile. Bladder ultrasonography revealed vesical thickening and intravesical clot deposits, evocative of haemorrhagic cystitis. The patient declined cystoscopy and received empirical treatment with furadantine 50 mg target dose for 5 days.

In November, she presented left-knee arthritis, recurrence of haemorrhagic cystitis and bilateral conjunctivitis. The right knee was clinically normal. Arthrocentesis showed an inflammatory liquid (leukocyte count 12,600/mm^3^) with no crystals and negative Gram stain. Cultures remained sterile; broad-range 16 S ribosomal RNA PCR of synovial fluid was negative. Reactive arthritis was considered, but urine samples were negative for *Chlamydia trachomatis, Neisseria gonorrhoeae* and *Mycoplasma genitalium*. STI multiplex real-time PCR of a urine sample was positive for *U. parvum* on retrospective analysis (not done at that time). No antibiotics were administered, and the evolution was favourable without sequelae.

**Fig. 1:**
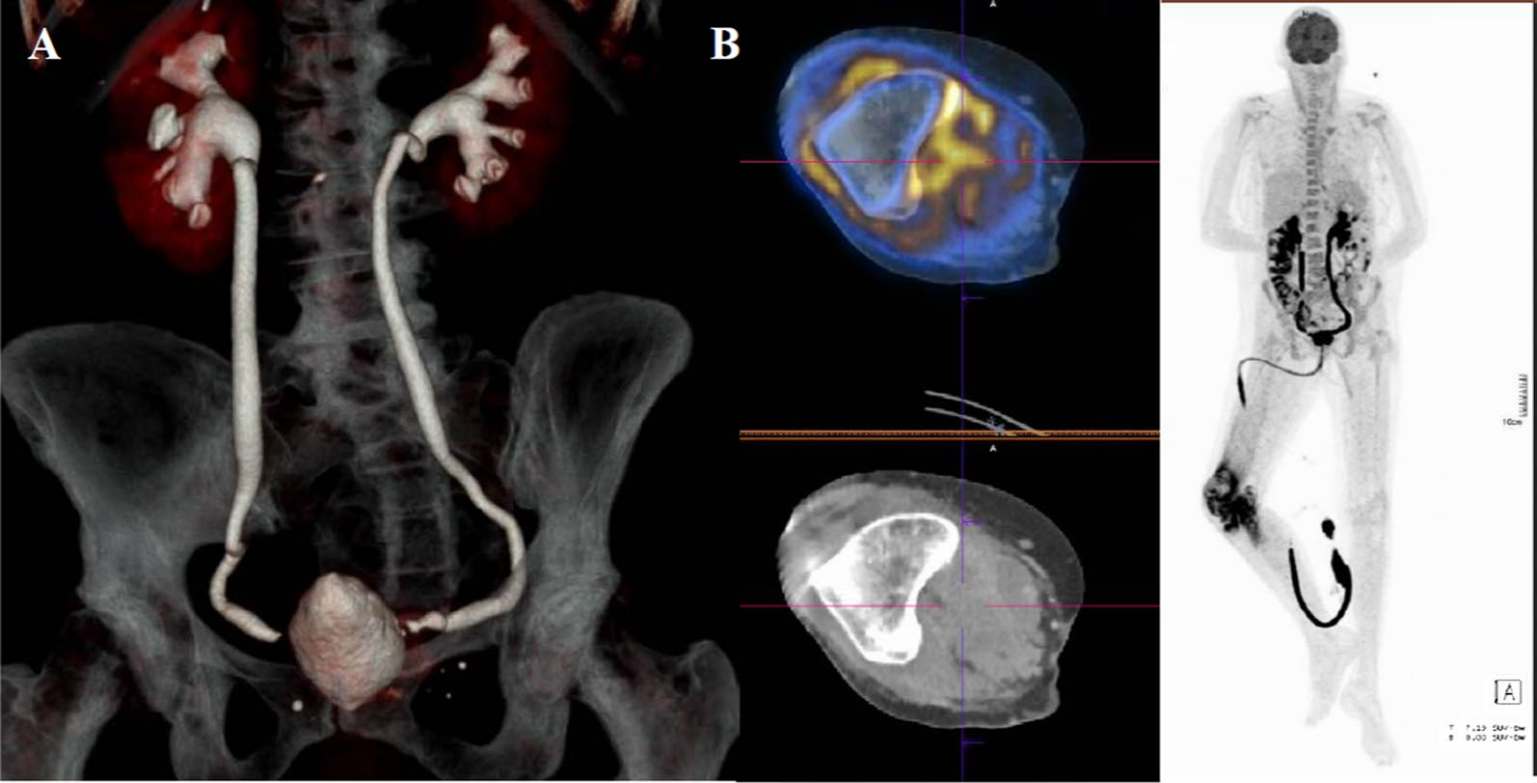
**a** Positron emission tomography tomodensitometry showing hypermetabolism of the right knee (maximal standardized uptake value [SUVmax]: 11.8) and right latero-urine hypertabolism (SUVmax: 7.5). **b** Computed tomography showing dilatation of urinary tract and bilateral ureteral stenosis

In March 2019, the cystitis worsened, and fever recurred. The patient was admitted with contralateral (right) knee arthritis and acute renal failure. The left-knee examination was unremarkable at that time. CT revealed cystitis and bilateral ureteral stenosis responsible for dilatation of the urinary tract (Fig. 1b). CT did not reveal any stone formation.

Double-J stents were placed and ceftriaxone was initiated. Blood and urine culture remained sterile and fever persisted. Right knee puncture retrieved purulent liquid, with leukocyte count 1620 000/mm^3^ (80% neutrophils, negative Gram stain). Then, hypotension developed: piperacilline-tazobactam was started, surgical knee drainage was performed, and intracellular arthritis was suspected. Multiplex STI real-time PCR (Allplex STI Essentials, Seegene), was strongly positive for *U. parvum* on synovial fluid and on a urine sample. Specific culture media was inoculated (A7agar, Biomérieux and MYCOFAST RevolutioN ElitechGroup), and A7 agar culture was positive for *U. parvum* in urine sample, but antibiotic susceptibility testing was negative. Bladder biopsy showed aspecific inflammation; PCR was negative for *U. parvum* although after 5 days of doxycycline treatment. 18 F-fluoro-2-deoxy-D-glucose positron emission tomography showed right-knee and right latero-uterine hypermetabolism (Fig. 1a), but the left knee was not hypermetabolic. Uterine echoendoscopy was non-contributive.

The diagnosis retained was *U. parvum* subacute ureterocystitis complicated by oculo-urethro-synovial syndrome (reactive arthritis) and subsequently by obstructive renal failure and septic arthritis. Antibiotic therapy with doxycycline 100 mg twice a day was started for 3 months. The fever receded, but local inflammation persisted. A control arthrocentesis after 15 days without antibiotics showed persistent arthritis but negative PCR results for *U. parvum*. Clarithromycin was introduced for 6 weeks, with marked clinical improvement: signs of erythema, swelling and pain disappeared, despite functional sequelae (flexion limitation 90°). Antibiotic treatment was well tolerated, and no clinical or biological event appeared during treatment.

## Discussion and conclusions

We report a case of right-knee reactive arthritis that was spontaneously regressive without sequelae. The right-knee arthritis preceded left-knee septic osteoarthritis associated with bilateral ureteral stenosis secondary to chronic ureterocystitis due to *U. parvum* infection.

*U. parvum* colonisation of the urogenital tract has been reported, and a high load of *U. parvum* has been found associated with chronic non-gonococcal urethritis in men, although whether it was a cause or a marker of non-gonococcal urethritis was unclear [9]. Currently, the paucity of documented *U. parvum* infection suggests opportunistic pathogenicity. Indeed, most infections have been reported in immunosuppressed or post-surgical patients. Ureaplasma are a common observation in patient with B-cell specific dysfunction such as patients with Common Variable Immune Deficiency [10]. For example, the first *U. parvum* prosthetic joint infection [11] was reported in 2014 in a patient withhypogammaglobulinemia, obesity and diabetes. Native joint infections have often been reported as part of systemic infections: bilateral knee arthritis with aortic co-infection by *U*. *parvum* and *Mycoplasma hominis *[12] as well as fatal mycotic aneurysms was reported in a 54-year-old man with lymphoma. Korytny et al. reported *U. parvum* orchitis complicated by gleno-humeral arthritis and endocarditis in a 56-year-old man with lymphoma history [13]. However, isolated chronic genitourinary infection by *U. parvum* has never been reported.

In the present case, retrospective assessment of the November 2018 STI multiplex real-time PCR of a urine sample was positive for *U. parvum*. However, given the frequency of genital colonization [8] and the usual absence of pathogenicity of *U. parvum*, this result was not considered significant, and only *C. trachomatis*, *N. gonorrhoeae* and *M. genitalium* results were reported. In the absence of other causal pathogens, clinical presentation and evolution pointed towards a diagnosis of *U. parvum* cystoureteritis at that time. Hence, we suggest that testing for *U. parvum* in subacute cystitis/ureteritis should be considered in immunosuppressed patients after excluding usual (including intracellular) pathogens and mycobacterial sampling.

*Ureasplama sp*. are not observed on Gram staining, and cultures are only contributory for high bacterial load and require specific media. Moreover, classical identification techniques may require 3 to 7 days, whereas real-time PCR is faster and more sensitive for *Ureaplasma* detection [14]. Use of next generation sequencing (NGS) is also a valuable technique to detect microbial cell-free DNA [15] and sometimes even if antibiotic was used [5]. Therefore, PCR should be combined with specific media culture and NGS should be considered. As ureaplasma is deprived of cell walls, antibiotics of the beta-lactam group are not effective. Conversely, doxycycline and clarithromycin are highly effective antibiotic, with low rates of resistance reported. About 7.5 % *U. parvum* are resistant to doxycycline by carrying the resistance gene tet(M) [16].

In our case, overlooking *U. parvum* as a possible etiology for the reactive arthritis delayed the diagnosis and allowed septic complications to develop. Indeed, in November, the patient presented left-knee arthritis associated with cystitis and conjunctivitis (oculo-urethro-synovial syndrome). Direct examination, culture and 16 S real-time PCR of arthrocentesis fluid gave negative results. This episode receded in the absence of antibiotic treatment. Together with spontaneous healing, the association of conjunctivitis and ureterocystitis with a sterile arthritis suggested reactive arthritis to *U. parvum* ureterocystitis.

Reactive arthritis is a rare immune-mediated arthritis following a genitourinary or gastrointestinal infection in predisposed patients and corresponds to the classic clinical triad: urethritis, eye disorders and arthritis. The principal microorganism involved in genitourinary infections responsible for reactive arthritis is *C. trachomatis*, but *Salmonella spp., Shigella spp., Campylobacter jejuni* and *Yersinia spp*. are also frequently involved. *Chlamydophila pneumoniae, Clostridium difficile*, HIV and *U. urealyticum *[4] have been described as uncommon inciting agents of reactive arthritis.

To our knowledge, we report the first case of oculo-urethro-synovial syndrome due to *U. parvum* ureterocystitis in an immunosuppressed patient.

In immunosuppressed people, arthritis can be caused by mycoplasma species. Cultures of *U. parvum* are not relevant, and PCR analysis is the best way to diagnosis the infection. PCR analysis of synovial liquid should be performed for immunosuppressed patients, especially in the presence of gynaecologic or urinary symptoms. *U. parvum* is also likely responsible for reactive arthritis.

It did not need formal ethics approval as this complies with national guidelines (“Loi n° 78 − 17 du 6 janvier 1978 relative à l’informatique, aux fichiers et aux liberté and « Règlement (UE) 2016/679 du Parlement européen et du Conseil du 27 avril 2016, relatif à la protection des personnes physiques à l’égard du traitement des données à caractère personnel et à la libre circulation de ces données ») .

## Supplementary Information

**Additional file 1.** Timeline.

## Data Availability

Materials analysed during the current study are not publicly available because of patient privacy concerns but are available from the corresponding author on reasonable request.

## Abbreviations

STI: Sexually transmitted disease
R-CHOP: Rituximab, cyclophosphamide, doxorubicin, vincristine, and prednisone
