# Supplementary figures and images for: Dual Ureaplasma parvum arthritis: a case report of U. parvum septic arthritis following contralateral reactive arthritis in an immunosuppressed patient
Source: BMC Infect Dis. 2021 Oct 29;21:1117. doi: 10.1186/s12879-021-06733-0 (PMC8556906; doi:10.1186/s12879-021-06733-0)

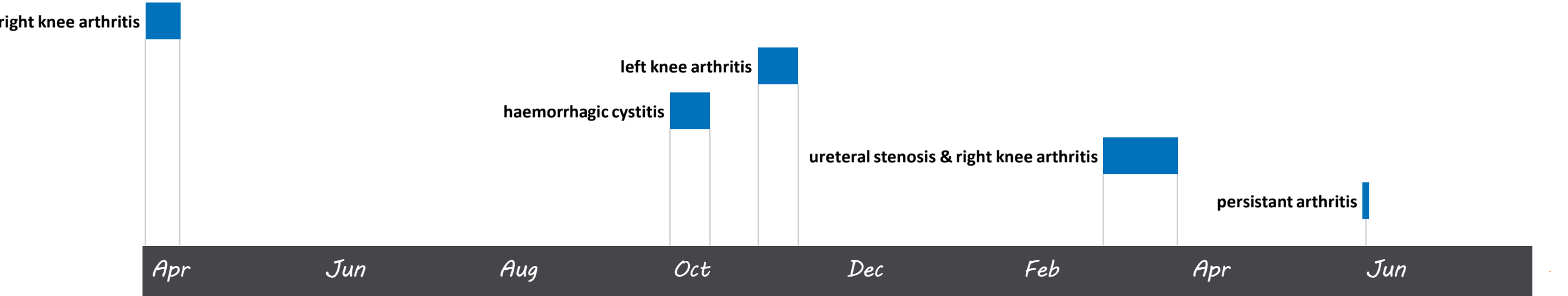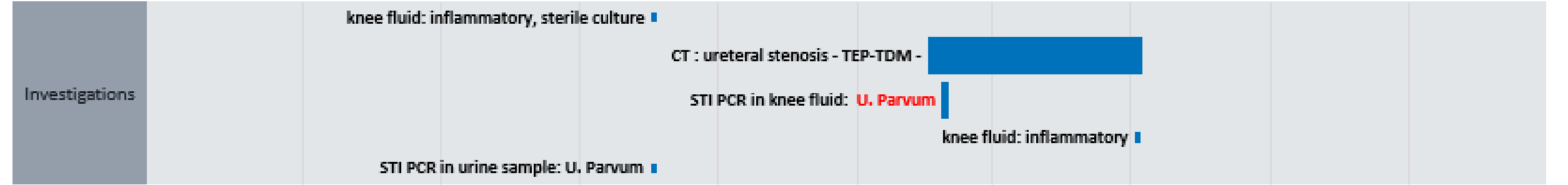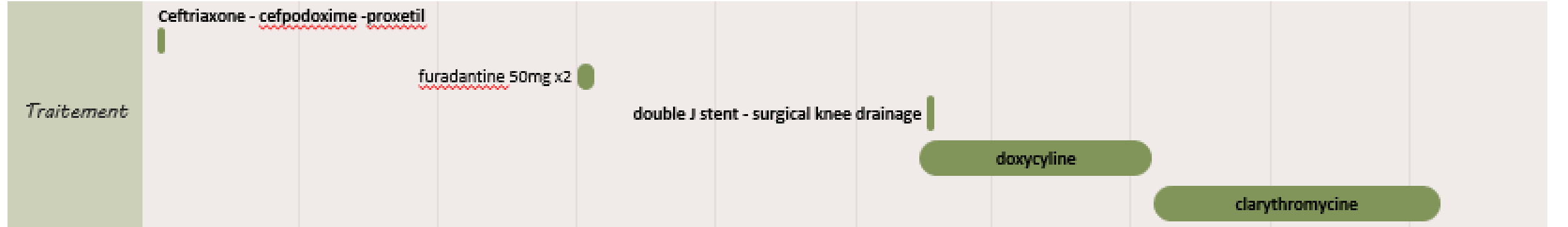

Supplement: Supplementary file 1 — Additional file 1. Timeline. [file 12879_2021_6733_MOESM1_ESM.pdf]
